# Involvement of Smad7 in Inflammatory Diseases of the Gut and Colon Cancer

**DOI:** 10.3390/ijms22083922

**Published:** 2021-04-10

**Authors:** Edoardo Troncone, Irene Marafini, Carmine Stolfi, Giovanni Monteleone

**Affiliations:** Department of Systems Medicine, University of Rome “Tor Vergata”, 00133 Rome, Italy; troncone.edoardo@gmail.com (E.T.); irene.marafini@gmail.com (I.M.); carmine.stolfi@uniroma2.it (C.S.)

**Keywords:** Crohn’s disease, ulcerative colitis, inflammatory bowel diseases, TGF-beta

## Abstract

In physiological conditions, the human intestinal mucosa is massively infiltrated with various subsets of immune cells, the activity of which is tightly regulated by several counter-regulatory factors. One of these factors is transforming growth factor-β1 (TGF-β1), a cytokine produced by multiple cell types and targeting virtually all the intestinal mucosal cells. Binding of TGF-β1 to its receptors triggers Smad2/3 signaling, thus culminating in the attenuation/suppression of immune–inflammatory responses. In patients with Crohn’s disease and patients with ulcerative colitis, the major human inflammatory bowel diseases (IBD), and in mice with IBD-like colitis, there is defective TGF-β1/Smad signaling due to high levels of the intracellular inhibitor Smad7. Pharmacological inhibition of Smad7 restores TGF-β1 function, thereby reducing inflammatory pathways in patients with IBD and colitic mice. On the other hand, transgenic over-expression of Smad7 in T cells exacerbates colitis in various mouse models of IBD. Smad7 is also over-expressed in other inflammatory disorders of the gut, such as refractory celiac disease, necrotizing enterocolitis and cytomegalovirus-induced colitis, even though evidence is still scarce and mainly descriptive. Furthermore, Smad7 has been involved in colon carcinogenesis through complex and heterogeneous mechanisms, and Smad7 polymorphisms could influence cancer prognosis. In this article, we review the data about the expression and role of Smad7 in intestinal inflammation and cancer.

## 1. Introduction

The human intestine contains more immune cells than the rest of the body and this is because a myriad of dietary and microbial antigens continuously stimulate the intestinal immune system. The small intestine and colon contain many Peyer’s patches and isolated lymphoid follicles, and B lymphocytes, plasma cells, T lymphocytes, natural killer cells, dendritic cells (DC) and macrophages infiltrate the lamina propria compartment. T cells and innate lymphoid cells (ILCs) are also present in the gut epithelial compartment [1,2]. This state of “low-grade inflammation” provides resistance to invading pathogens without altering the digestive functions and tissue integrity [3]. Several counter-regulatory factors restrain the activity of the effector immune cells, thereby contributing to maintain gut homeostasis. Studies in animal models of immunity and inflammation indicate that reduced expression/function of such factors can break the immune tolerance towards the luminal microflora, thereby resulting in chronic colitis [2,4,5,6,7,8,9]. One such factor is Transforming Growth Factor (TGF)-β1, a component of the TGF-β superfamily [10]. In mammals, there exist three isoforms of TGF-β: TGF-β1, 2 and 3. TGF-β2 and TGF-β3 mainly play a role in bone development and muscle, while TGF-β1 is involved in the differentiation and functions of many immune cells, including T and B lymphocytes, NK cells and DCs [11].

TGF-β1 signals through two transmembrane receptors with serine/threonine kinase activity, named TGF-β1 Type 1 Receptor (TβR1) and TGF-β1 Type 2 Receptor (TβR2) [12]. TGF-β1 binds TβR2, thus leading to auto-phosphorylation of the receptor and subsequent recruitment of TβR1, with the formation of a transmembrane heterodimer. TβR2 phosphorylates TβR1 and, then, the activated TβR1-TβR2 complex promotes phosphorylation of Smad2 and Smad3, two proteins that interact with Smad4 to form a complex, which moves to the nucleus to regulate the transcription of several genes [12,13]. TGF-β1 can also activate other pathways, such as AKT/PI3K and MAP kinases [14]. In the gut, epithelial cells and several immune cells, particularly DCs, are important sources of TGF-β1. TGF-β1 is secreted as an inactive form bound to the latency-associated peptide that masks its binding site to TGF-βR2. Activation of the cytokine needs conformational changes or protein cleavage of the latency-associated peptide by several molecules, such as metalloproteinases, furin and the integrins αvβ6 and αvβ8 [15].

**Figure 1:**
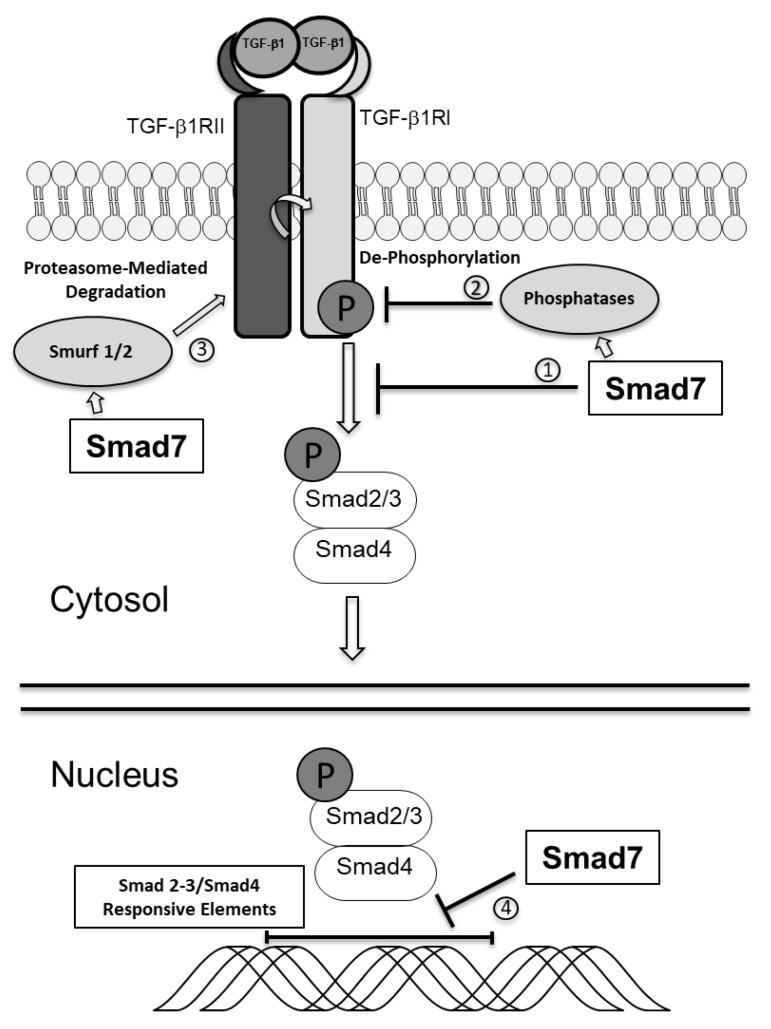
Smad7 negatively regulates TGF-β1 signaling through various mechanisms: 1. binding to TβR1 prevents Smad2/3 phosphorylation; 2. de-phosphorylation and inactivation of TβR1 through recruitment of phosphatases; 3. interaction with E3 ubiquitin ligases SMURF1/2 to promote proteasome-mediated degradation of TβR1; 4. in the nucleus, through inhibition of the association of the Smad2-3/Smad4 complex with specific Smad-responsive DNA sequences.

Another Smad protein, termed Smad7, negatively regulates TGF-β1-associated Smad signaling through various mechanisms (Figure 1). For instance, Smad7 can bind to TβR1 and prevent Smad2/3 phosphorylation [16,17]. Smad7 can also facilitate recruitment of phosphatases to TβR1, thereby promoting de-phosphorylation and inactivation of the receptor [18], and promote proteasome-mediated degradation of TβR1 in cooperation with SMURF1/2, two E3 ubiquitin ligases [19,20]. Smad7 can also localize into the nucleus and limit the association of the Smad2-3/Smad4 complex with specific Smad-responsive DNA sequences [21]. Smad7 can also exert some regulatory effects on the expression and function of molecules controlling inflammatory and neoplastic processes in a TGF-β1-independent manner [22].

We here review the available evidence supporting the involvement of Smad7 in intestinal inflammation and colon cancer (CC).

## 2. Smad7 in Human IBD

Crohn’s disease (CD) and ulcerative colitis (UC) are the major human inflammatory bowel diseases (IBD) [23]. In CD, the inflammation is transmural and segmental and can involve any part of the gastrointestinal tract, but lesions are more frequent in the distal part of the small intestine and right colon. In contrast, UC-associated inflammation involves the mucosa, and the lesions arise in the rectum and can extend proximally to the whole colon [23]. Both CD and UC can associate with the development of local complications and extra-intestinal manifestations. The etiology of both IBD is unknown but it has been supposed that interaction between environmental and genetic factors triggers an excessive immune reaction within the intestinal wall, which ultimately leads to a tissue-damaging pathological process [24]. Defects of counter-regulatory factors/mechanisms contribute to amplify the IBD-associated immune–inflammatory response. One such a defect involves the TGF-β1/Smad2/3 signaling pathway. In the normal intestinal mucosa, lamina propria mononuclear cells (LPMC) constitutively express phosphorylated Smad3, clearly indicating that endogenous TGF-β1 works properly. Indeed, culturing normal intestinal mucosal explants with a blocking TGF-β1 antibody increases expression of inflammatory proteins (i.e., T-bet and IFN-γ), which are known to be negatively regulated by TGF-β1 [25]. Moreover, recombinant TGF-β1 inhibits NF-kB activity and, hence, inflammatory cytokine synthesis in LPMC isolated from the normal colonic mucosa [26]. In contrast, IBD LPMC are resistant to the TGF-β1-mediated immune suppression, as evidenced by no change in NF-kB activation and production of inflammatory cytokines following stimulation with recombinant TGF-β1 [26]. In line with this, IBD LPMC exhibit reduced levels of phosphorylated Smad2/3 as compared to normal LPMC [27]. Overall, these findings are consistent with the demonstration that intestinal samples taken from inflamed areas of IBD patients contain elevated levels of the inhibitory Smad7 [27]. Immunohistochemical analysis of inflamed tissue sections of IBD patients revealed that Smad7 is up-regulated in both epithelial cells and LPMC [28]. By assessing mucosal samples taken from patients with established CD and post-operative recurrence, we also showed that induction of Smad7 occurs very early in the inflammatory cascade, which leads to the mucosal damage [29]. Evaluation of Smad7 RNA and protein in paired IBD mucosal samples showed that up-regulation of Smad7 occurs at the protein level but not at the RNA level, thus suggesting a post-transcriptional regulation of Smad7 in IBD [30]. By using several approaches, we also showed that IBD biopsy samples contain reduced levels of ubiquitinated Smad7 as compared to normal control samples, while in CD and UC, there is acetylation of Smad7, a dynamic post-translational modification, which makes the protein resistant to proteasome-mediated ubiquitination-driven degradation [30]. This latter phenomenon is, at least in part, due to the activity of the transcriptional coactivator p300, which is over-expressed in inflamed areas of IBD patients and promotes Smad7 acetylation [30]. Smad7 is also regulated by SIRT1, a member of the mammalian Sirtuin family, which deacetylates the lysine residues of Smad7, thus reducing Smad7 content. In IBD mucosa, SIRT1 expression is reduced, particularly in Smad7-expressing cells [31]. 

## 3. Evidence Supporting the Pathogenic Role of Smad7 in the Gut

The demonstration that Smad7 is over-expressed in IBD tissue concomitantly to the reduced TGF-β1/Smad2/3 signaling prompted us to mechanistically evaluate the involvement of Smad7 in the IBD-associated immune–inflammatory response. Smad7 inhibition in IBD mucosal explants and LPMC with a specific Smad7 antisense oligonucleotide (AS) enhances Smad3 phosphorylation and reduces synthesis of inflammatory cytokines. A neutralizing anti-TGF-β1 antibody blocks the Smad7 AS-mediated anti-inflammatory effects [27].

TGF-β1 promotes, both directly and indirectly, differentiation of T regulatory cells (Tregs), which express the transcription factor Foxp3 and exert regulatory functions by acting mainly on effector CD4+ T cells [32,33]. CD4+ T cells isolated from the inflamed gut of CD patients are resistant to Tregs-mediated suppression and this phenomenon is dependent on Smad7, since Smad7 knockdown makes CD4+ T cells responsive to Tregs [34].

There is also preliminary evidence supporting the role of Smad7 in the positive regulation of CCL20, a chemokine involved in the recruitment of immune cells to the intestine and over-expressed in the CD epithelium [35]. Indeed, in CD, CCL20 and Smad7 co-localize, and AS-induced Smad7 knockdown in CD explants reduces CCL20 production [35].

Up-regulation of Smad7 also occurs in the colon of mice with oxazolone-induced colitis and mice with trinitrobenzene sulfonic acid (TNBS)-induced colitis, two animal models showing morphological and immunological similarities with UC and CD, respectively [36,37,38]. In both these models, the elevated levels of Smad7 associate with reduced Smad3 phosphorylation, and colitic mice given oral Smad7 AS show enhanced phosphorylation of Smad3, reduced synthesis of inflammatory cytokines and amelioration of colitis [38]. 

To further assess the pathogenic role of high Smad7 on the ongoing colitis, Fantini et al. generated a transgenic (Tg) mouse over-expressing Smad7 in T cells and NKT cells [34]. Those mice do not spontaneously develop colitis but they are more susceptible to dextran sodium sulphate (DSS)-induced colitis [39]. Moreover, by using the T cell transfer model of colitis, it has been shown that the adoptive transfer of Smad7 Tg naïve CD4+ T cells, in the absence of Tregs, into naïve mice induces a colitis that is more severe than that documented in mice reconstituted with wild-type T cells [34]. Smad7 Tg T cells exhibit decreased content of aryl hydrocarbon receptor (AhR), a transcription factor that enhances IL-22 synthesis and induces regulatory responses in the gut [40]. Consistently, in normal but not in IBD LPMC, TGF-β1 enhances AhR expression [41]. In the T cell transfer colitis model, AhR activation ameliorates the course of colitis induced by wild-type T cells but does not influence colitis induced by Smad7 Tg T cells [41]. Although these findings underline the pathogenic role of Smad7 in T cells, little is known about the specific functions of Smad7 in other immune cells and non-immune cells in the gut. In this context, however, Garo and colleagues recently showed that specific deletion of Smad7 in DCs, as well as in CD4+ T cells, enhances TGF-β1 responsiveness and attenuates experimental colitis in mice [42]. Analysis of the molecular mechanisms by which Smad7 mediates intestinal inflammation showed that Smad7 deficiency leads to increased programmed death (PDL)2/1-PD1 signaling. PPD1 and its ligands PDL1 and PDL2 negatively regulate effector T cell responses and, therefore, are critical in the maintenance of immune tolerance [43,44,45]. PDL1 and PDL2 are expressed on antigen-presenting cells, including DCs, while PD1 is expressed on CD4+ T cells. Engagement of PDL1 on DCs by PD1 promotes Foxp3+ Treg differentiation [46,47]. Consistently, DC-specific Smad7 deletion mitigated DSS-induced colitis by inducing CD103+PDL2/1+DC and Tregs [42]. Similarly, colitic mice intraperitoneally given Smad7 AS exhibit attenuation of DSS-induced colitis and increased TGF-β1 and PDL2/1-PD1 signaling [42]. 

CD can be complicated by the development of fibrostrictures, the most common indication for surgical resection in this disorder [48]. The pathogenesis of such a complication is not fully understood, but experimental evidence suggests that TGF-β1/Smad signaling regulates various steps of the fibrogenic process. To assess the role of Smad7 inhibition in the development of colitis-driven intestinal fibrosis, Izzo et al. used the mouse model of TNBS-mediated colitis-driven intestinal fibrosis [49]. Oral administration of Smad7 AS to colitic mice reduced the degree of colitis and attenuated the degree of intestinal fibrosis [49].

In the gut, the epithelial layer acts as physical barrier protecting the host from the luminal content [50]. The intestinal epithelial barrier is maintained through a complex system of junctions, which include tight and adherent junctions. Alterations or defects of such a system lead to increased permeability and eventually intestinal inflammation [51]. TGF-β1/Smad signaling has a central role in epithelial homeostasis and wound repair and enhances epithelial barrier protection against intestinal pathogens [52,53]. Moreover, mice lacking intestinal expression of Smad4 exhibit increased colonic permeability and a significant decrease in several claudin proteins [54]. It has also been demonstrated that the active form of vitamin D, 1,25-dihydroxyvitamin D3, increases claudin-4 levels by blocking Smad-7 activity in mucosal samples from active UC, thus contributing to restoring the impaired epithelial function in these patients [55]. These findings, although not conclusive, suggest Smad7 as a possible target to restore TGF-β1-dependent epithelial barrier integrity and function in intestinal inflammatory disorders. 

## 4. Smad7 in Other Inflammatory Pathologies of the Gut

Celiac disease is a gluten-driven immune-mediated disease of the small intestine characterized by various degrees of villus atrophy and symptoms/signs of malabsorption [56]. Treatment of celiac disease is based on exclusion of gluten from the diet, which results, in most patients, in restoration of the normal mucosal architecture and resolution of symptoms/signs [57]. However, in a small fraction of patients, the symptoms/signs of malabsorption and villous atrophy can persist or recur despite a strict gluten-free diet. This condition is termed refractory celiac disease (RCD) and associates with an elevated risk of complications [58,59]. The pathogenesis of RCD is not fully understood but many inflammatory cytokines produced in the inflamed gut of these patients are supposed to amplify the tissue-destructive immune response [60]. The Smad7 protein, but not RNA, is over-expressed in the duodenum of RCD patients as compared to active and inactive celiac disease patients and normal controls, and this associates with reduced phosphorylation of Smad2/3 [61]. Knockdown of Smad7 with AS in ex vivo organ cultures of RCD duodenal samples reduces inflammatory cytokine production, supporting the pathogenic role of this protein in the RCD-associated cytokine response [61].

Increased expression of Smad7 has also been seen in the gut of patients with necrotizing enterocolitis (NEC), an inflammatory bowel necrosis of premature infants [62]. Macrophages in surgically resected tissue samples of NEC patients, but not in the uninflamed mucosa of controls, show strong cytoplasmic and nuclear immunoreactivity for Smad7, mostly in areas with severe tissue damage. Up-regulation of Smad7 also occurs in a murine model of NEC where gut injury is inducible in formula-fed mice by hypoxia and hypothermia [62]. Mechanistically, bacterial products induce Smad7 expression in neonatal macrophages; Smad7, in turn, sensitizes macrophages to bacterial products and promotes NF-κB activation and cytokine production. Smad7 induces IKK-β expression in macrophages through direct binding and transcriptional activation of the IKK-β promoter. Notably, IKK-β enhances Smad7 expression, thereby setting up a positive feedback loop, which could contribute to amplifying the inflammatory activation of macrophages in NEC [62]. In line with this, studies in preterm baboons, which are at risk of NEC due to deficiency of TGF-β2 in the developing intestine, show increased expression of Smad7 in the normal preterm intestinal epithelial cells and during NEC. It was also shown that Smad7 binds to the TGF-β2 promoter, thereby suppressing TGF-β2 expression [63].

**Figure 2:**
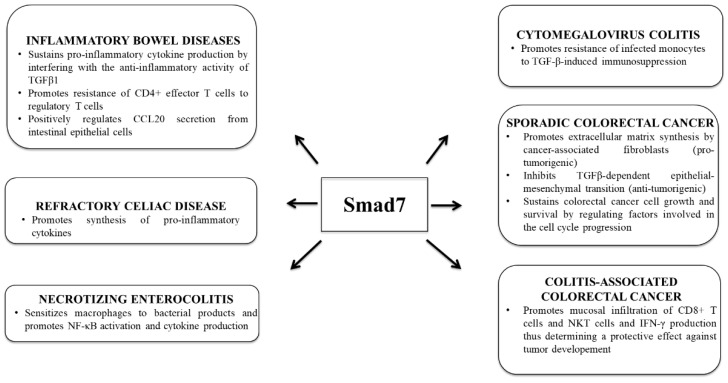
Function of Smad7 in intestinal inflammatory diseases and colon cancer.

The gastrointestinal mucosa is a major site of opportunistic cytomegalovirus (CMV) infection, which causes local inflammation and, in some circumstances, severe end-organ dysfunction [64,65,66]. Concurrent CMV can also worsen the course of IBD and promote severe flare-up of the disease. It is well-known that, after primary infection, CMV typically persists in the bone marrow, infecting progenitor myeloid cells, which then enter the circulation as latently infected monocytes [67,68]. The latter cells can be recruited into the intestinal lamina propria, where they differentiate into infected macrophages secreting high levels of pro-inflammatory cytokines [69,70,71,72]. CMV infection of monocytes enhances expression of Smad7, thus blocking the ability of TGF-β1 to inactivate NF-κB activation and NF-κB-dependent cytokine production [73]. Consistently, Smad7 is over-expressed in mucosal tissues from patients with CMV-induced colitis and declines after antiviral ganciclovir therapy [73]. Knockdown of Smad7 with AS in CMV-infected monocytes restores monocyte susceptibility to TGF-β-induced immunosuppression [73]. The role of Smad7 in various inflammatory diseases and cancer is summarized in Figure 2.

## 5. Smad7 in Colon Cancer

CC is one of the most common causes of cancer-related morbidity and mortality worldwide [74]. In more than two thirds of cases, CC arises as a sporadic disease, while in 2–3% of cases, CC complicates the natural history of IBD (colitis-associated cancer, CAC) with a cumulative risk depending on disease duration, extension and severity of the inflammatory lesions [75,76]. Reilly and colleagues recently examined the association between single-nucleotide polymorphisms (SNPs) in the Smad7 gene and CC development in 90 patients with UC [77]. Genotyping was performed for the *Smad7* rs4464148, rs11874392, rs12953717 and rs4939827 SNPs. None of such *Smad7* SNPs were associated with the development of UC-associated CC at an allelic or genotypic level [77]. Rizzo et al. recently showed that the number of Smad7-positive CD4+ T lymphocytes in the inflamed mucosa of IBD complicated by CAC is diminished as compared to that seen in the mucosa of uncomplicated IBD [39]. In a murine model of CAC induced by azoxymethane and DSS, Smad7 Tg mice develop a severe colitis characterized by a massive infiltration of the mucosa with CD8+ T cells and NKT cells and increased production of IFN-γ. However, those mice develop less tumors than control mice. The latter protective effects are dependent on IFN-γ, as genetic deletion of such a cytokine abolishes the beneficial effect of Smad7-over-expressing T cells on CAC formation [39]. Along the same line is the demonstration that Smad7 Tg mice develop less tumors than wild-type mice following the subcutaneous injection of syngenic MC38 colon carcinoma cells [78].

Cancer-associated fibroblasts (CAFs) represent a large proportion of the cell population within the tumor microenvironment, and their presence in the stroma associates with poor prognosis in CC, given the ability of such cells to produce growth factors and cytokines that promote CC cell proliferation and immune evasion, to deposit and remodel the extracellular matrix (ECM) and, hence, to generate a growth-supportive desmoplastic stroma [79,80,81]. In contrast to the normal conditions, where activation of fibroblasts is a reversible phenomenon, CAFs remain reprogrammed and pathologically activated throughout tumor progression [82]. It is thus plausible that, under normal conditions, there exist factors/mechanisms, which switch off fibroblast activation, while this regulatory control is likely lost during tumorigenesis. Recent studies indicate the involvement of type I interferon (IFN1) in this process. IFN1 biological activity is mediated by a heterodimeric receptor complex composed of IFNAR1 and IFNAR2 chains [83]. Proteolytic loss of IFNAR1 in the nonimmune tumor microenvironment promotes a stable activation of CAFs and enhances tumor growth, whereas stabilization of the receptor attenuates the pro-tumorigenic phenotype of CAFs [84]. Mice deficient in IFNAR1 exhibit enhanced expression of Smad7 in fibroblasts, and knockdown of Smad7 in such cells alleviates deficient ECM production in response to TGF-β1 [84]. These data raise the possibility that Smad7 is one of the mediators of the IFN1-driven suppression of stromagenesis. 

A different scenario emerges from studies investigating the role of Smad7 expressed by cancer cells [85]. Human CC specimens express high levels of Smad7 in both cancer cells and LPMC and CC cell lines contain much more Smad7 than non-tumoral intestinal epithelial cell lines. AS-mediated knockdown of Smad7 reduces the in vitro and in vivo proliferation of CC cells [22]. The anti-mitogenic effect of Smad7 AS relies on modulation of some cell cycle-related proteins, which eventually induces cells to arrest in the S phase of the cell cycle [22]. Specifically, inhibition of Smad7 induces phosphorylation of eukaryotic translation initiation factor 2 α (eIF2α), a transcription factor involved in the regulation of cell cycle arrest, and consequently up-regulation of activating transcription factor 4 (ATF4) and CCAAT/enhancer binding protein homology protein (CHOP) [86]. eIF2α phosphorylation and ATF4/CHOP induction are secondary to enhanced activity of serine–threonine protein kinase RNA (PKR) as its silencing abrogates the Smad7 AS effect on the cell cycle [86]. 

In CC tissue, there is a strong positive correlation between Smad7 and circTBL1XR1, a circRNA that enhances the malignant and metastatic behavior of CC cells [87]. Moreover, Smad7 expression in CC tissue associates with significantly short overall survival and disease-free survival of patients [88]. 

SNPs of the Smad7 gene have been associated with sporadic CC [89,90]. In particular, three SNPs of the Smad7 gene, rs4939827, rs12953717 and rs4464148, were identified in genome-wide association studies by Broderick et al. for both adenomas and CC [91]. Carriers of the rs4939827 homozygote variant genotype show a 27% reduced risk of CC, while rs12953717 and rs4464148 are associated with a 37% and 35% increased risk, respectively, for those with the homozygote variant genotype. Evaluation among individuals with a family history of CC documented an inverse association between the rs4939827 *Smad7* SNP and CC [91]. In contrast, Tenesa and colleagues documented a statistically consistent 20% increased risk of CC for the rs4939827 *Smad7* SNP [92]. A further genome-wide association study in a large population-based case–control study of CC showed that rs12953717 associates with a statistically significant increased risk of CC for the TT genotype compared with the CC genotype, whereas the CC genotype of the rs4939827 SNP is inversely associated with CC relative to the TT genotype [93]. A meta-analysis including 63 studies and 187,181 subjects documented an association between rs4939827, rs4464148 and rs12953717 variants and CC [90]. A study investigating susceptibility loci, which could predict rectal cancer prognosis after surgery, confirmed that the rs12953717 and rs4464148 variants are significantly associated with rectal cancer recurrence and patients with lower Smad7 expression exhibit a longer disease-free survival [94]. Using biopsy samples of 264 CC patients, Boulay and colleagues showed that deletion of *Smad7* is less frequent than that of *Smad4* and of *Smad2*, and patients with deletion of *Smad7* have a significantly better prognosis than patients with two copies of this locus. On the other hand, amplification of *Smad7* associates with a significantly worse prognosis, suggesting the oncogenic role of Smad7 in sporadic CC [95]. 

During epithelial–mesenchymal transition (EMT), epithelial cells lose apical–basal polarity with disturbance of cell-to-cell tight contacts and the cytoskeleton and acquire a mesenchymal phenotype, which facilitates transformed epithelial cells to resist apoptosis, invasion and migration [96,97,98]. Wang and colleagues showed that nuclear reporter subfamily 2, group F, and member 2 (NR2F2), also known as COUP TF II or ARP2, is up-regulated in a variety of cancers, including CC. NR2F2 inhibits Smad7 expression and promotes TGF-β-dependent EMT of CC cells via transactivation of miR-21 [99]. 

Taken together, the above findings suggest that Smad7 can play divergent roles in the function of the cell types involved in CC with the downstream effect of either inhibiting or accelerating the different phases of colon carcinogenesis.

## 6. Open Questions and Future Perspectives

The findings discussed here highlight the role of Smad7 in amplifying signals which contribute to the progression of IBD and other inflammatory pathologies of the gut. In patients with such disorders, the abundant expression of Smad7 associates with reduced TGF-β1-mediated immune suppression, and knockdown of Smad7 allows the endogenous TGF-β1 to become functional and inhibit detrimental signals. These observations paved the way for the development of an oral Smad7 AS-containing pharmaceutical compound, initially named GED0301 and later on mongersen [100]. Phase 1 and phase 2 studies documented clinical and endoscopic benefits of mongersen in active CD patients [100,101]. However, a more recent phase 3, multicenter, double-blind, placebo-controlled study was discontinued in October 2017 as an interim analysis documented a lack of efficacy of the drug [102]. The reasons for the discrepancy between phase 2 and phase 3 studies are unknown. In this context, it has been recently demonstrated that some batches of the drug used in the phase 3 study were not able to inhibit Smad7 expression in the CC cell line HCT-116 [103]. Conversely, other batches of mongersen, which down-regulated Smad7 in HCT-116 cells, were effective in inducing a clinical response and remission in active CD patients [103]. Further work is needed to ascertain whether and which physical/chemical modifications, which could occur at the large-scale synthesis of the antisense oligonucleotides, may have disrupted the pharmacological activity of some batches used in the phase 3 program. 

We are also evaluating whether Smad7 is preferentially induced in some of the evolutive phases of CD and ascertaining whether molecular profiling of IBD patients could help identify the better candidates for the treatment with mongersen. Since the *Smad7* rs144204026 C/T SNP maps on the corresponding region targeted by the Smad7 AS contained in mongersen, Di Fusco et al. examined whether such a variant allele affects the ability of Smad7 AS to knockdown Smad7. Initially, no TT genotype in CD patients as well as in healthy volunteers was found. In contrast, a heterozygous genotype was more frequent in CD patients as compared to controls. Smad7 AS down-regulated Smad7 RNA independently of the presence of the variant allele, thus excluding the possibility that carriers of such a variant are not good candidates for mongersen treatment [104]. In this context, it is noteworthy that a recent study has documented an association between the rs12956924 variant within the Smad7 gene and UC in a Japanese cohort [105]. 

Some aspects of the expression and function of Smad7 in IBD and in CC remain to be investigated. For instance, it remains unclear whether in these pathologies there is a cell-specific regulation of Smad7 and which factors contribute to induce Smad7 in the specific cell types. As pointed out above, in IBD, Smad7 is up-regulated in epithelial cells but we do not yet know if, in such cells, Smad7 positively regulates inflammatory pathways and/or phases of the mucosal healing process. We also need to ascertain whether and which regulatory effects of Smad7 on the intestinal inflammation and colon carcinogenesis are independent of TGF-β1 and dissect further mechanisms of action of Smad7. It also remains unclear if expression of Smad7 in either IBD tissue or peripheral blood may help identify specific subsets of patients. Indeed, it has been shown that, before initiation of anti-TNF and after 2 weeks of treatment, the expression of Smad7 in whole blood samples of IBD children was decreased in patients who were considered as non-responders compared to responders [106]. Although this study deserves confirmation, the findings suggest that Smad7 can be a potential pharmacogenomic marker for early response to TNF blockers in pediatric IBD patients. 

## 7. Conclusions

A large body of evidence indicates that Smad7 can influence the delicate balance between anti-inflammatory and pro-inflammatory signals in the gut, thus promoting detrimental inflammatory responses. Smad7 inhibition in IBD patients with an oral Smad7 AS-containing pharmaceutical compound showed conflicting clinical results, and further studies are ongoing to possibly confirm the efficacy of such approach in clinical practice. Furthermore, the broad variety of intestinal disorders in which a role of Smad7 has been hypothesized and/or demonstrated opens multiple possibilities about future therapeutic approaches. Among these, Smad7 modulation for CC therapy remains an intriguing but still open topic, due to the extremely complex role of Smad7 in cancer biology. These fascinating fields will represent objects of future research.

## Data Availability

Not applicable.
